# Urinary Incontinence Secondary to Fecal Impaction in a Patient With Likely Undiagnosed Hirschsprung’s Disease

**DOI:** 10.7759/cureus.16837

**Published:** 2021-08-02

**Authors:** Simon Kashfi, Carlos Sermeno Camacho, Karen L Castro

**Affiliations:** 1 Internal Medicine, City University of New York (CUNY) School of Medicine, New York, USA; 2 Pediatrics, St. Barnabas Hospital Health System, Bronx, USA

**Keywords:** hirschsprung's disease, urinary incontinence, spinal cord compression, constipation, meconium

## Abstract

Hirschsprung’s disease is caused by the failure of migration of neural crest cells to the hindgut, causing a lack of development of ganglion cells in the submucosal and myenteric plexuses of the colonic wall. Hirschsprung’s disease most often presents in infants with failure to pass meconium in the first two days of life. We present the case of an eight-year-old male with chronic constipation since birth who presented to the emergency department with signs concerning spinal cord compression. To our knowledge, this is the first of such a case presenting with urinary incontinence.

## Introduction

Hirschsprung’s disease occurs in 1/5,000 live births with a male to female ratio of 3:1-4:1 [1]. It is caused by the failure of migration of neural crest cells into the hindgut [2]. This failure causes a lack of development of ganglion cells in the submucosal and myenteric plexuses of the colonic wall [3]. The defect is most often confined to the rectum and sigmoid colon [3,4]. The gold standard for diagnosing Hirschsprung's disease is a full-thickness rectal suction biopsy, which allows for the evaluation of aganglionosis of the submucosal and myenteric plexuses [3]. About 80% of patient’s Hirschsprung’s disease are diagnosed in the first few months of life, with up to 90% failing to pass meconium before 24 hours of life [4,5]. Those who present later in life often have a prolonged history of constipation that is managed by the pediatrician with laxatives and stool softeners [1,5-8]. We present the case of an eight-year-old male with chronic constipation since birth who presented to the emergency department with signs concerning spinal cord compression. To our knowledge, this is the first of such a case presenting with urinary incontinence.

## Case presentation

The patient is an eight-year-old male with a history of chronic constipation since birth who presented to the emergency department after an episode of urinary incontinence that he did not feel. He was in his usual state of health until five days ago when he started to complain of pain in the posterior left thigh. The pain progressed over several days and became excruciating, radiating from the buttock to the back of his knee. The intensity of the pain awoke him from sleep. He also endorsed numbness of the left big toe. Trials of Motrin and naproxen at home did not improve his pain.

Upon further questioning, pregnancy was uneventful, and the patient passed meconium soon after birth. His parents had been managing his constipation with over-the-counter suppositories at least once a month. A trial of polyethylene glycol did not help. At baseline, he has a bowel movement once or twice every two weeks. His last bowel movement was six days ago, and it was hard and small. His mother also reports that he has daily encopresis.

On physical exam, he was in excruciating pain. His abdomen was distended, with diffuse, mild tenderness to palpation. He walked with an antalgic gait. Tenderness to palpation over the left buttock was noted. Patellar reflex was 2+, and gastrocnemius reflex was 1+. Pulses were 2+ through the left lower extremity, and the sensation was notable for being slightly decreased in the left big toe.

**Figure 1:**
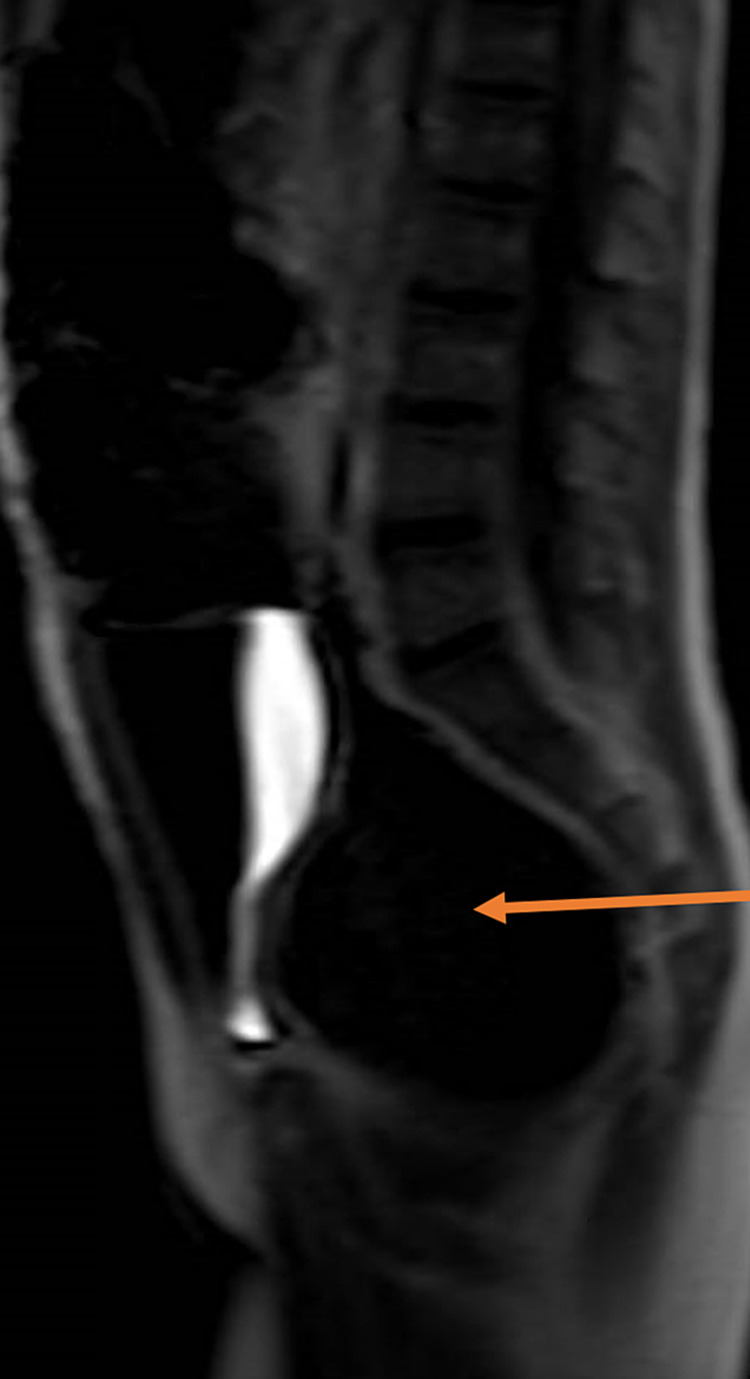
Fecal mass (arrow) compressing the bladder.

At this time, the differential diagnosis was broad but suspected spinal cord compression and neuromuscular disorder were the most concerning, and thus pediatric neurology was consulted. They recommended an MRI of the spine to rule out spinal cord compression. Results showed marked distension of the rectosigmoid colon with intrinsic mass, likely fecal impaction, partially compressing the urinary bladder (Figure 1). The mass measured approximately 7.9 x 6.5 x 10 cm in size. It was concerning for short segment megacolon, possibly due to Hirschsprung’s disease. The patient was transferred to another hospital for pediatric gastroenterology evaluation. There, he underwent fecal decompaction under anesthesia, followed by bowel cleanout. MRI of the left leg from hip to knee was within normal limits. The patient was also evaluated by neuromuscular medicine. EMG study revealed very mild, possible motor axon loss in the left fibular nerve, but it was not significant when compared to the asymptomatic right leg. His symptoms were most consistent with lumbosacral plexopathy, primarily affecting the L5 and fibular segments. It was thought that this could have been due to compression from his fecal impaction.

Finally, the patient underwent rectal suction biopsy. Unfortunately, the biopsy was limited to the level of the muscularis mucosa, and no submucosal tissue was seen. Thus, the biopsy was unable to evaluate for aganglionosis. The patient's symptoms had improved after fecal decompaction, and his leg pain had resolved. As of now, there are no plans for repeat biopsy as the parents would like to pursue alternative therapies.

## Discussion

Hirschsprung’s disease has been reported to be diagnosed as late as 67 years of age [1]. Some authors classify Hirschsprung’s disease after 10 years of age as adult Hirschsprung's [9], but it is unclear as to whether this is due to late-onset or failure of diagnosis [6]. A systematic review of 490 cases of adult Hirschsprung’s disease showed that almost 79.8% of patients had disease confined to the rectum, and 12.5% of patients had recto-sigmoid disease [6]. Based on the data here and our imaging, we presume that the patient had some form of the short-segment disease [3]. These presentations would be classified as a short-segment disease [3]. Long segment disease would extend through the sigmoid colon, and total aganglionosis would affect the whole colon. A rare form, known as ultra-short segment disease, is confined just to the distal rectum [3,5]. Patients with delayed onset of Hirschsprung’s disease have had bowels that were able to compensate, though up to a certain point. Additionally, there is usually a history of delayed passage of meconium in patients with Hirschsprung’s diagnosed after infancy [3,7].

Of note, our patient presented with signs concerning spinal cord compression. The radiating buttock pain and urinary incontinence raised concern for cauda equina syndrome, prompting immediate spinal imaging. Interestingly, sciatica has been reported as a consequence of large fecal impaction, with pain relief after decompression [10]. The EMG results do suggest nerve compression secondary to fecal impaction. Additionally, to our knowledge, our patient is the first case reported in the literature of urinary incontinence secondary to fecal impaction. This could have been due to the mass compressing on the bladder, as shown in Figure 1. It could also have been due to compression of the nerves leading to the bladder. A different case has shown ureter and iliac vessel compression due to fecal impaction, which ultimately caused lower leg edema [11].

## Conclusions

Hirschsprung’s disease most often presents in children with delay or failure to pass meconium. In some cases, fecal impaction can be so severe that it compresses intra-abdominal structures. In this case, the impaction caused sciatica and urinary incontinence, concerning spinal cord compression. To our knowledge, this is the first case in the literature to present both symptoms. Pediatricians should be aware that this disease may go undiagnosed during infancy and consider possible workup for patients with chronic constipation.
